# Advances in Functionalization of Bioresorbable Nanomembranes and Nanoparticles for Their Use in Biomedicine

**DOI:** 10.3390/ijms241210312

**Published:** 2023-06-18

**Authors:** Ahammed H. M. Mohammed-Sadhakathullah, Sofia Paulo-Mirasol, Juan Torras, Elaine Armelin

**Affiliations:** 1 Departament d’Enginyeria Química, EEBE, Universitat Politècnica de Catalunya, C/Eduard Maristany, 10-14, Ed. I.2, 08019 Barcelona, Spain; ahammed.hussain.madhani@upc.edu (A.H.M.M.-S.); sofia.paulo@upc.edu (S.P.-M.); 2 Barcelona Research Center for Multiscale Science and Engineering, Universitat Politècnica de Catalunya, C/Eduard Maristany, 10-14, Ed. I.S, 08019 Barcelona, Spain

**Keywords:** bioresorbable polymers, nanomembranes, nanoparticles, biomedical devices, biomedical implants, drug delivery

## Abstract

Bioresorbable nanomembranes (NMs) and nanoparticles (NPs) are powerful polymeric materials playing an important role in biomedicine, as they can effectively reduce infections and inflammatory clinical patient conditions due to their high biocompatibility, ability to physically interact with biomolecules, large surface area, and low toxicity. In this review, the most common bioabsorbable materials such as those belonging to natural polymers and proteins for the manufacture of NMs and NPs are reviewed. In addition to biocompatibility and bioresorption, current methodology on surface functionalization is also revisited and the most recent applications are highlighted. Considering the most recent use in the field of biosensors, tethered lipid bilayers, drug delivery, wound dressing, skin regeneration, targeted chemotherapy and imaging/diagnostics, functionalized NMs and NPs have become one of the main pillars of modern biomedical applications.

## 1. Introduction

The development of polymer-based biomedical devices has been aided by the discovery of a new class of materials known as bioresorbable polymers. The term “bioresorbable” has become a widely used phrase to describe this class of macromolecules. The following is a scientifically accepted definition for such materials: a material for which the degradation is mediated, at least partially, from a biological system [1]. Systems made of bioresorbable polymers degrade naturally in the human body; the main advantage is that they do not need to be removed, representing a reduction in surgery complications, helping thus to maximize patient comfort and safety. On the other hand, the rejection of absorbable implants is also a risk to be taken into account, as well as the possible toxicity of the degradation products [2] of said biodegradation. Therefore, bioresorbable polymers must present high biocompatibility and non-toxicological effects belonging to either the polymer itself or the degraded products [3,4]. Thus, in vivo biocompatibility assays of any new bioresorbable material is of utmost relevance [5].

Currently, there are numerous types of bioresorbable polymeric materials with different fabrication, functionalization, and application mechanisms that are being introduced in many fields of human life [6]. Examples of their chemical composition can be seen in Table 1. The most employed biopolymers belong to polysaccharides compounds such as chitin and chitosan and proteins such as collagen and gelatin [7]. Special mention deserves to bacteria-derived polymers such as polyhydroxyalkanoates (PHA), which usually exhibit non-inflammatory or immune responses in vivo [8]. On the other hand, synthetic bioresorbable polymers belong to petrochemical feedstock monomers with controlled and high purity, and, therefore, they are often preferred for the fabrication of grafts, prostheses, and other biomedical tools compared to biopolymers. Included in this category, the most important classes are polyesters (poly(glycolic acid) (PGA) [9], poly(lactic acid) (PLA) [10,11], and poly(ε-caprolactone) (PCL) [12]) and polyethers such as poly(ethylene glycol) (PEG), even if some of them can be obtained or produced by enzymes bio-based [13]. This development continually extends to envelop more areas, and the direction is slowly moving to target suitable bioresorbable soft materials for specific applications such as tissue regeneration [14,15,16], temporary prostheses [17,18,19], drug carriers [20,21,22], biosensing [23], cancer therapy [24], and others.

Among the applications of such a particular class of bioresorbable compounds, nanomembranes (NMs) and nanoparticles (NPs) (and microspheres) are two of the most common forms being explored. Biological NMs are naturally functionalized systems with channel proteins, essentially present in all living organisms. Artificial NMs comprise biomimetic systems that are multi-functionalized to partially or fully mimic the barrier protection and specific transport properties of natural biological membranes. Either NMs or NPs have thicknesses and diameters, respectively, in the nanometric scale. For instance, a variety of biological systems are well-known as natural NPs for their small dimensions such as vesicles, lipoproteins, magnetosomes, viruses, etc. [25]. NMs can be defined as soft or hard structures with the upper thickness limit of 100 nm (theoretically) [26], and about 2–3 nm (<10 nm) of thickness in the case of cell membranes. NPs can be considered as nanospheres if their diameters range from 10–20 nm to 500 nm as maximum dimension (theoretically), whereas microspheres have micro-dimensions of 500–1000 nm in diameter or higher (giant structures) [22,27].

**Table 1:** Summary of different polymeric families and their biomedical applications.

Types of Bioresorbable Polymers	Polymers	Chemical Structure	Applications	Refs.
Natural Macromolecules				
Polysaccharides	Chitosan	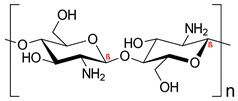	Nano delivery systems, antibacterial agent, wound healing, tissue regeneration	[7,28,29]
	Hyaluronic acid	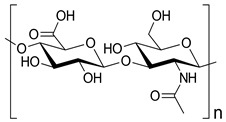	Drug delivery, bone regeneration, and wound dressing	[30,31,32]
	Alginic acid	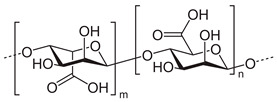	Drug delivery, wound healing, tissue engineering, cell transplantation, gene therapy, environmental remediation, developing bioanalytical markers	[33,34]
	Chondroitin sulphate	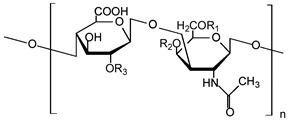	Anticoagulant, articular cartilage repair, corneal lesion healing, antidiabetic, and antiproliferative effects	[35,36,37,38]
	Dextran	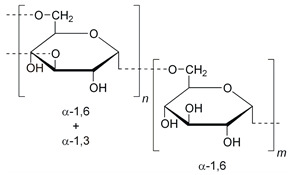	Anticoagulant, antiviral drugs, in vivo imaging, non-fouling surfaces, and anti-HIV (human immunodeficiency virus) agents	[39,40,41,42,43]
	Agarose	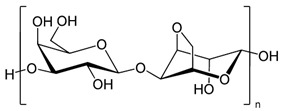	Drug delivery systems, wound healing, tissue engineering, enzyme immobilization, and regenerative medicine	[44,45,46,47,48]
Proteins	Collagen	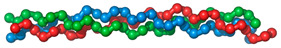 Main structural protein of tissues	Haemostatic agent, wound dressing, tissue engineering, and reconstructive medicine	[49,50,51,52]
	Gelatin	Mixture of peptides and proteins produced by partial hydrolysis of collagen	Food packaging, gene, drug and cell delivery	[53,54,55,56]
	Elastin	Protein encoded as ELN gene	Dermal regeneration, synthetic vascular grafts, and tissue regeneration	[57,58,59]
	Silk	Natural protein fiber	Biosensors, drug delivery systems, tissue engineering, cell adhesion, cancer therapy, and implant coatings	[60,61,62,63,64,65]
	Fibrin	Fibrous, non-globular protein	Tissue regeneration, drug delivery, wound repair, tissue support, gluing, and sealing	[66,67]
Polyester	Shellac	Complex resin, mainly composed of aleuritic acid, jalaric acid, shellolic acid, and other natural waxes	Microencapsulation, food coatings, drug delivery, tissue engineering	[68,69,70,71]
Synthetic macromolecules				
Homopolymers	Poly (lactic acid) (PLA)	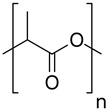	Nano and micro particulate delivery systems, intradermal implant, sutures, biomolecules immobilization, imaging, and bone tissue engineering	[72,73,74,75]
	Poly (glycolic acid) (PGA)	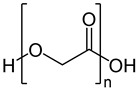	Drug release, operative assist, damage healing, suture, reinforcement, and scaffold	[76,77]
	Poly-ε-caprolactone (PCL)	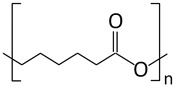	Drug delivery systems, tissue regeneration, vascular grafts, dental implant coating, wound dressing, and stem cells enrichment	[12,21,78,79,80]
	Poly(vinyl alcohol) (PVA)	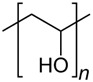	Antibacterial, tissue engineering, drug carrier, and wound dressing	[81,82]
Copolymers	Poly(lactic-co-glycolic acid) (PLGA)	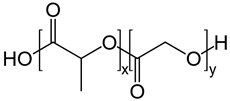	Nano and micro particulate delivery, bone and tissue engineering, wound healing, vascular grafts, chemotherapy, cancer diagnosis, and imaging	[52,83,84,85,86,87,88,89,90]
	Poly((S)-3,3-dimethylmalic acid) (PDMMLA)	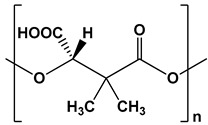	Coating for endovascular stents, drug delivery systems, and implants	[91,92,93,94]
	Poly(p-dioxanone) (PPDO)	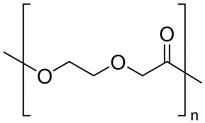	Sutures, bone, or tissue repair and drug delivery systems	[95,96,97,98]
	Poly(propylene fumarate) (PPF)	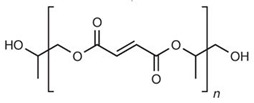	Bone tissue engineering, vascular graft, drug delivery, bone cement, soft tissue adhesives, and cardiac patches.	[99,100,101,102,103]
	Polyanhydrides	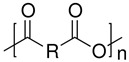	Cargo delivery and polyprodrugs	[104,105,106,107,108,109]
	Polycarbonates	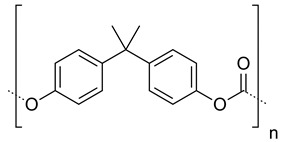	Drug release, gene delivery, vascular prostheses, soft tissue engineering, and cell culture scaffolds	[104,110,111,112,113,114,115,116,117,118,119,120]
	Polyurethanes	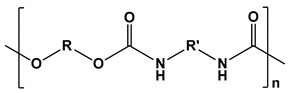	Wound dressings, artificial organs, vascular stents, tissue engineering, drug delivery, short-term implants, and scaffolds	[121,122]
	Poloxamers	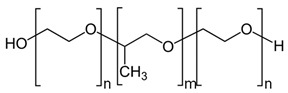	Tissue surfactants, drug release, medical device coatings, tissue engineering, cancer therapy, and theranostic platforms	[123,124,125,126]
	Poly(ortho esters)	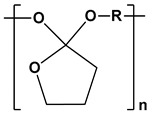	Gene delivery, drug delivery, and tissue engineering	[127,128]
	Polyalkylcyanoacrylates	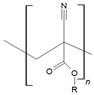	Drug delivery and oxygen carriers for blood substitutes	[129,130]
	Polyamides	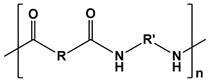	DNA delivery and tissue engineering	[131,132,133,134,135,136,137,138]
	Polyhydroxyalkanoates (PHAs)	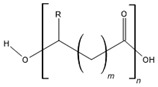	Soft and hard tissue engineering and drug delivery	[8,139]
	Polyphosphazenes	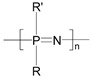	Stent coatings, immunoadjuvant, DNA and drug delivery, and tissue engineering	[140,141,142,143]
	Polyethers	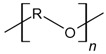	Tissue engineering, delivery devices, and scaffolds	[144,145,146,147,148]

NMs have become incredible consideration in the last decades due to their exceptional characteristics such as high flexibility, stability, robustness, and molecular permeability along with tunable pore size, shape, and densities [149]. This comes about in the wide range of applications within biotechnology and biomedicine as they are utilized as sensors [150,151], biomotors [152], bio-interfaces for cellular frameworks [153,154,155], antimicrobial surfaces [156,157], and drug release devices [158,159,160], as stated before. In addition, the surface of the NMs can be altered using other molecules such as proteins, drugs, and fluorescent probes, which broaden the applicability of these materials for different medical fields such as wound dressing, tissue engineering, and health care monitoring [161].

**Figure 1:**
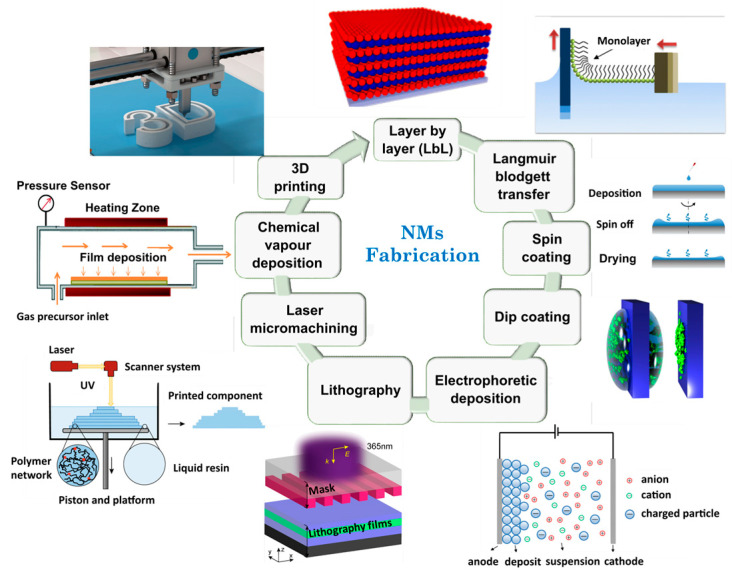
Illustration of main fabrication methodologies for NMs obtaining. Adapted from References [166,167,168,169,170,171]. Copyright 2016 Royal Society of Chemistry, 2019 American Chemical Society, and 2021 Elsevier.

In this way, several approaches have been detailed within the literature for synthesizing NMs [149,161,162]. Layer-by-layer (LbL) assembly [163], Langmuir–Blodgett transfer (LB) [164], spin-coating [155,162], dip coating [161], electrophoretic deposition, and cross-linking of self-assembled monolayer (SAM) techniques [165] are some examples of techniques employed for film preparation and functionalization (Figure 1).

On the other hand, functional polymeric micro- and nanospheres, which are commonly employed in chemical catalysis and adsorption systems such as drug delivery [172], have completely different methods for their obtaining than that exemplified for NMs. Those systems are characterized to have a large specific surface area, high adsorption capacity, surface reaction ability, and ease of polymerization method application (aqueous, organic, or water/oil media) [173]. In addition, control over particle size, homogenous distribution, and particle isolation are the most important drawbacks that can hinder reproducibility and good yields over the production processes.

For two decades, the most prominent field of NPs applications has been in the pharmacy, as a drug carrier. The utilization of synthetic biodegradable polymers as microspheres in this area, as well as in NMs applications, has exponentially increased. PLA [174], poly(lactic-co-glycolic acid) (PLGA) [86], and PCL [78] are some of the degradable polymers that can be utilized for this purpose. Polyester copolymers or blends, composed of PLA and other degradable segments (PLGA, PCL, PHA), have been widely investigated due to their enhanced biodegradability and tunable mechanical properties [73]. They can be developed and synthesized with a variety of molecular weights and lactide:glycolide ratios for specific purposes, as in the case of PLGA, while maintaining excellent reproducibility and minimal cost [172].

**Figure 2:**
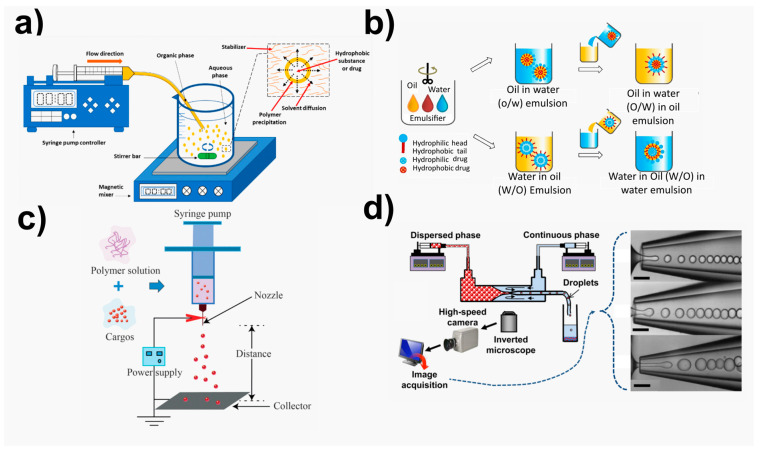
Illustration of main fabrication methodologies for NPs obtaining. (**a**) Nanoprecipitation methods, adapted from ref. [175]. Copyright 2016 MDPI. (**b**) Emulsion-based methods. Adapted from ref. [172]. Copyright 2016 Elsevier. (**c**) Electrosprayed carriers, adapted from ref. [176]. Copyright 2019 Frontiers. (**d**) Microfluidic methods, adapted from ref. [177]. Copyright 2015 American Chemical Society.

The most widely used and promising methods for forming nano-sized particles (Figure 2) can be categorized into four groups, as detailed by Lee et al. [172]: (i) traditional emulsion-based technologies such as single emulsion, double emulsion, and multiple emulsions; (ii) nano-precipitation, fast expansion of supercritical fluid into liquid, salting out, and dialysis are all examples of precipitation-based procedures; (iii)direct compositing processes such as melting, spray drying, supercritical fluid, and in situ generating micro-particles; (iv) new approaches that include microfluidic techniques and template/mold-based techniques, exemplified for PLA polymer [11,78,172,174].

This review summarizes the main methods currently carried out for the functionalization of NMs and NPs made with synthetic biodegradable polymers for medical purposes, along with a thorough discussion of their benefits and drawbacks. The key objective is to review the most recent applications in the last five years and describe the new features that are expected to be implemented in the context of these systems’ potential future applications. In contrast to earlier studies, this one provides a fresh perspective on the present landscape of bioresorbable materials research. Potential uses of these biomaterials that have not received much attention yet are highlighted including anchoring lipid bilayers, actively targeted chemotherapeutics, and theranostics.

## 2. Current Chemical and Physical Modifications of Nanomembranes and Nanoparticles to Afford Specific Functions

In the following section, an overview of surface functionalization methods for NMs and NPs is provided. These functionalization procedures make it possible to introduce reactive functional groups (–COOH, –OH, –NH_2_), conjugate bioactive compounds (polysaccharides, peptides, proteins, antibody/antigens & polymers) and drugs, improve cell affinity and adhesion, as well as wettability and other properties, into the biomaterials of interest.

### 2.1. Nanomembranes Functionalization

The function of synthetic NMs would essentially be mechanical and defensive, similar to their biological counterparts if there were no extra capabilities. The majority of roles that biological NMs play are made possible by integrated protein structures that guarantee additional functionalities with respect to synthetic NMs. The main difference at this current point of development is that biological structures provide significantly more complex features than artificial ones, but at the cost of having to use a much smaller toolbox in terms of chemical composition, material options, operating temperature and humidity ranges, and functionalities. Artificial structures use far more rudimentary and flawed processes, but there are a lot more functionalization options available, a bigger variety of materials, and a broader spectrum of pure functions. The latter offers a variety of choices and routes not found in nature.

**Figure 3:**
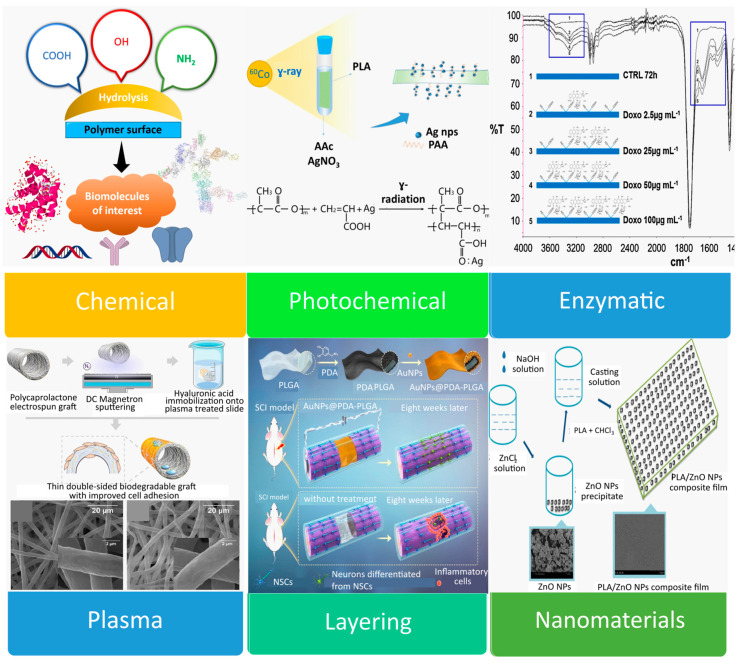
Illustration of the main NMs and NPs functionalization methodologies. Figures are adapted from references [74,89,195,196,197]. Copyrights 2019 American Chemical Society, 2017, 2020, 2022 and 2018 Elsevier, respectively.

There are two types of functionalization or modification strategies reported in the literature—bulk and surface. For the scope of this review, we adhere to surface modification techniques. Surface activation or surface modification refers to the application of some external method to a portion of or the entire surface of the nanomembrane to alter the chemical structure of its interfacial components that interact with the environment [178]. The applied alterations may just be found at the nanomembrane’s volume or they may extend throughout the entire surface area. Chemical [179,180,181], photochemical [182,183], enzymatic [74,184,185], and plasma treatment [186,187] are a few possible activation methods (Figure 3). In surface activation, boundary atoms or molecules may be removed or modified, surface bonds may be broken or created, polar groups may be created or destroyed, etc. The properties of the nanomembrane may undergo drastic alterations as a result of this surface activation, maybe changing by many orders of magnitude after the surface functionalization.

In the material that follows, we cover each of the relevant approaches to nanomembrane functionalization in more depth.

#### 2.1.1. Chemical

Among chemical treatments, alkaline surface hydrolysis is a straightforward method for generating reactive functional groups on polymers such as PLA, such as hydroxyls (–OH) and carboxylic acids (–COOH) [73]. With surface-modifying species containing amine (–NH_2_) or hydroxyl (–OH) groups, the resultant carboxylic acid groups can be easily coupled. Cai et al. added chitosan covalently to poly(D,L-lactic acid) (PDLLA) surface via acid-chitosan conjugation after alkaline surface hydrolysis (which produced acid groups). This PDLLA surface modification enhanced cell affinity exhibiting a great increase in rat osteoblast attachment and proliferation [188].

PLA model surfaces have been functionalized using elastin-like recombinamers (ELRs), recombinant engineered proteins [179]. After the films were treated with sodium hydroxide, N-(3-dimethylaminopropyl)-N′-ethylcarbodiimide (EDC), and NHS, amine-reactive coupling agents were used to react with them. The primary amino groups of biomolecules such as peptides, ELRs, and bovine serum albumin (BSA) were used to graft them together. According to the study, covalent bonding enhances cell adherence and dissemination when compared to samples that were physiosorbed.

Another method for adding reactive amine groups to polymer surfaces is aminolysis [189]. Aminolysis and conjugation with biocompatible macromolecules such as collagen, chitosan, or gelatin have been accomplished using 1,6-hexanediamine [190].

There are two widely used methods for manufacturing surface-tethered polymer surfaces which are known as the “graft-in” and “graft-from” methodologies. In the former, previously synthesized polymer chains are attached to a surface, while in the second technique, the surface is first functionalized with a monolayer of initiator followed by an ulterior growth of polymer brushes which are initiated directly from the surface [191]. It should be mentioned that both the functional group activation step and the polymer grafting step can be achieved by either chemical or photochemical activation. Several examples using chemical grafting [192] and photochemical grafting [193] methodologies have been reported elsewhere.

#### 2.1.2. Photochemical

Photochemical methods such as photografting have been widely utilized to modify the surface characteristics due to the benefits it provides, including the following: cheap operating costs, mild reaction conditions, UV light selectivity, and permanent surface chemistry alteration [182]. In this method, reactive groups are created using polymer photoactivation and then grafted with specific functionality.

Shen et al. [194] used photochemical grafting by UV light to modify poly(dimethyl)siloxane (PDMS) surface to be more resistant to cell adhesion. PDMS is well known to be prone to adsorption of both proteins and bacteria, thus favoring potential infection in implants using this material. Using a simple single-step photografting technique, poly(sulfobetaine methacrylate) (pSBMA) and poly(carboxybetaine methacrylate) (pCBMA) thin films were covalently attached to PDMS substrates. pCBMA-coated materials exhibited significant reduction in bacterial adhesion and growth with respect to *S. aureus* and *S. epidermidis* as well as the ability to resist bacterial growth on PDMS implants.

Recently, by simultaneously inducing grafting polymerization and Ag reduction with γ-ray radiation, PLA films with enhanced hydrophilicity and antibacterial characteristics were created by Qi et al. [195].

#### 2.1.3. Enzymatic

Around ten years ago, the initial findings on the bio-catalyzed activations of polyester-based materials sparked interest in the chemo-enzymatic functionalization of surfaces. In 2009, Donelli et al. [198] published a thorough study on the characterization of enzymatically activated poly (ethylene terephthalate) membranes, which included water-contact angle (WCA), Fourier-transformer infrared spectroscopy (FTIR), and fluorescence spectroscopy (FS). In this study, the free surface carboxylic groups produced by the enzymatic attack were coupled with fluorescent alkyl bromide-2-(bromo-methyl) naphthalene. This study emphasizes the way that enzymatic treatment enables the surface functionalization of extremely thin polymeric layers while preserving the bulk characteristics [198]. The principle of “limited surface hydrolysis” was created and applied to biodegradable polymers. This method was used as the activation step for the hydrophilization of the polymeric surface and the production of hydroxyl and carboxyl groups. In a subsequent step, several reactions were carried out including the enzymatic esterification of the hydroxyl groups with a fluorinated acid [184], the electrostatic coupling of the chemotherapy drug doxorubicin (and its subsequent controlled release in conditions that mimicked the physiological environment) [74], and the creation of a super hydrophobic material via the chemical coupling of a stearic-acid-based alkyl ketene dimer leading to a WCA > 150° [185]. Therefore, when modifying the outer layers of polymer films and microstructures is desirable but the capacity to maintain bulk properties is also required, the enzymatic functionalization of surfaces is an appealing strategy. Traditional methods such as severe chemical treatments such as NaOH, which would deeply damage the material’s molecular mass and mechanical characteristics, or plasma, which cannot reach the farthest corners and regions of complicated structures, may not otherwise be able to do this (i.e., the functionalization of the inner part of a cylindrical structure).

#### 2.1.4. Plasma Treatment

The mixture of positive ions and electrons created by ionizing the matter is known as “plasma”. In the past decade, surface hydrophilicity and cell affinity of polymers have improved thanks to plasma surface treatment, which was first used in the 1960s [199]. Hirotsu et al. [200] used oxygen, helium, and nitrogen plasmas to increase the wettability of melt-extruded PLA sheets. Yang et al. [75] improved the hydrophilicity and cell (human skin fibroblast) affinity of complicated forms such as porous PLA scaffolds made utilizing a particle leaching process by treating them with anhydrous ammonia (NH_3_) plasma. On PLA scaffolds, the NH_3_ plasma generated reactive amine groups that attached collagen via polar and hydrogen bonding interactions. These scaffolds with changed surfaces displayed improved cell adhesion [201]. PLA films have undergone an O_2_ plasma treatment to increase their wettability and nerve cell adhesion [202].

Materials with distinctive features are created when plasma treatment activates the polymer surfaces and forms a thin layer for the linker-free immobilization of bioactive molecules. By applying plasma treatment followed by hyaluronic acid immobilization, thin porous PCL-based double-sided grafts were created by Kudryavtseva et al. [196]. The suggested alteration enhances cell adherence without changing the morphological or mechanical characteristics of the graft. This method can be used on a variety of biodegradable polymers having hydrophilic inner surfaces and hydrophobic outer surfaces including PLA, polyglycolide, and their copolymers and mixes.

#### 2.1.5. Layering/Lamination

Making multilayered structures with each layer contributing its own features and functions is a process of functionalization known as lamination. In biological membranes, nature frequently employs this technique. It is also the simplest functionalization method, yet it is still quite flexible. Both the extra functionalities and the number of levels are completely discretionary [26].

The layered NMs may have two strata (the simplest case) or more. The nanocomposite’s multifunctionality is enhanced by the unique features and capabilities that each stratum introduces. One layer might be a plasmonic waveguide and ensure the possibility of biological sensing; another might be a layer with biological or chemical activity such as a ligand layer to attract only a particular species selectively; the next layer might be added to ensure the mechanical strength and robustness of the entire structure [168].

For example, Molina et al. [158] reported a novel method for producing flexible, robust, and electroactive free-standing multilayered films that combines in situ electropolymerized poly(3,4-ethylene-dioxythiophene) (PEDOT) and PLA, which offers mechanical strength. The conducting polymer PEDOT used in this work is often used to create biomedical devices because of its exceptional electrochemical characteristics, biocompatibility, and stability under continuous usage. The 5-layered films’ outstanding performance as bioactive platforms for the proliferation of fibroblast and epithelial cells was demonstrated in cell cultures, and their potential as selective bioadhesive surfaces for the separation of proteins was demonstrated in adsorption studies. A similar technique that successfully combines the mechanical benefits of free-standing (also known as self-supported) PLA ultrathin films and the electrochemical responsiveness of anodically polymerized CPs was used to create effective multilayered Faradaic motors [203]. It is also possible to create covalently bonded hydrogel layers on the surface of polymer films. The reactive groups were introduced by altering the surfaces of such films by means of an initial surface carboxylic groups activation using 1-hydroxybenzotriazol. A free-radical copolymerization technique, or the cross-linking of natural polymers, was used to create the hydrogel covering. In the first case, acrylamide copolymerization with N,N′-methylene-bis-acrylamide was used, whereas in the second, alginate cross-linking with L-lysine in the presence of water-soluble carbodiimide was used [204].

In a recent study, a unique three-layer nanomembrane structure was produced by immobilizing gold NPs to the PLGA membrane using polydopamine (PDA) [89]. The hydrophilicity and conductivity of the AuNPs@PDA-PLGA nanomembrane were excellent, and it had a good capacity for adsorbing proteins and for antioxidant activity. On the AuNPs@PDA-PLGA nanomembrane, electrical stimulation dramatically accelerated axon regeneration and growth and further enhanced neural stem cell proliferation and differentiation. The nanomembrane created in the procedure, however, lacks topographical elements that would direct the alignment of the neurons’ neurites.

#### 2.1.6. Nanomaterials Incorporation

The mechanical properties, selectivity, and permeability of the polymeric nanomembrane have changed significantly as a result of the incorporation of inorganic or organic nanomaterials into the matrix by creating a hybrid material surface. This has given researchers more opportunities to create applications that are more suitable and effective for biomedical purposes. For example, nanofiber membranes often contain metallic oxides, semiconductors, minerals, metals, and metal alloys (titanium dioxide (TiO_2_), zinc oxide (ZnO), silver (Ag), and silica (SiO_2_) NPs). Zinc oxide NPs, for instance, exhibit amazing mechanical and antibacterial properties when incorporated into PLA film [197]. Titanium dioxide (TiO_2_) NPs can also be added to the polymeric matrix to create nanocomposites with enhanced functionality [205]. It is now well acknowledged that adding NPs to a polymeric matrix is an easy way to enhance the material’s optical, mechanical, thermal, biomedical, electrical, barrier, and biodegradability, and broaden its uses.

### 2.2. Nanoparticles and Microspheres Functionalization

The most widely used polymers for NPs preparation are chitosan, alginate, gelatin, PLA, PGA, PLGA, poly(alkyl cyanoacrylates) (PACA), and PCL, which are known for both their biocompatibility and resorbability through natural pathways. Polymers NPs have gained interest due to their intrinsic characteristics to be used in drug delivery, biosensors, imaging, and sensing. The advantages of functional polymeric microspheres, which are commonly employed in chemical catalysis and adsorption, include a large specific surface area, high adsorption capacity, surface reaction ability, and ease of surface modification [206,207].

As we mentioned in the previous section, the most well-known methods to fabricate micro/nano-sized particles are (i) nanoprecipitation, (ii) spray drying, (iii) emulsion aqueous/organic (W/O), and (iv) microfluidic technique [177]. As the aim of this review is to explore in detail the functionalization of NPs, as well as to explore their potential applications in biomedical fields such as diagnostic agent [207], drug delivery [208], therapeutic agent [209], etc., we will not focus on the NPs fabrication. NPs have been functionalized to modify their properties improving their sensitivity and selectivity towards some specific target, as well as to enhance their specificity to work in complex matrixes.

NPs surface modification can be performed during the synthesis or post-treatment process that is performed by integrating various organic and inorganic molecules at the nanoscale level. Such integration can be conducted through the use of covalent and non-covalent bonds (i.e., hydrogen bonds and electrostatic and van der Waals interactions).

#### 2.2.1. Covalent Conjugation

Covalent conjugation functionalization is based on the attachment of a specific linker with a specific functional group on the NPs surface by covalent bonding. The use of cross-linkers depends on the functional group of the biomolecules and the NPs. The specific functional elements (peptide, protein, antibody/antigen) are covalently linked to the surface functional groups (–COOH, –NH_2_, and –N_3_) [81,210]. Several protocols have been developed using bifunctional cross-linkers in order to immobilize a specific biomolecule on top of the surface or just coupling agents to covalently link the functional element. The most common cross-linkers among other are glutaraldehyde (GA) and (3-aminopropyl)-triethoxysilane (APTES), being 1-ethyl-3-(3-dimethylaminopropyl) carbodiimide (EDC), and N-hydroxysuccinimide (NHS) one of the most used coupling agents through an stable amide bond formation. This functionalization method presents a rigid and stable formation compared to non-covalent conjugation.

In 2018, Raudszus et al. [210] published a fabrication of poly(vinyl alcohol) (PVA)-PLA NPs to be used as a platform to detect biomolecules in solution by means of emulsion-diffusion techniques. The PVA used as stabilizer was modified with divinyl sulfone to facilitate surface fuctionalization by apolipoprotein E, penetranin or ovalbumin. The use of a vinyl sulfone-modified poly(vinyl alcohol) (VS-PVA) derivative as a reactive steric stabilizer allows for efficient surface modification with amine- or thiol-containing ligands such as proteins and peptides. Prepared NPs showed a good biocompatibility and viability towards brain endothelial cells by WST-1-assay.

In 2022, Viana et al. [211] published the fabrication of protein–polymer conjugated NPs of BSA and thermoresponsive copolymer of N-vinylcaprolactam (NVCL) and N,N-dimethylaminoethyl methacrylate (DMAEMA) with excellent properties to be used in the biomedical field such as drug delivery, and also showing antitumor properties. BSA-P(NVCL-co-DMAEMA) nanoparticles were produced by controlled radical polymerization (CRP) employing grafting-from in aqueous medium using Cu(0)-mediated radical polymerization. They published a biocompatible platform that exhibits cytotoxicity against breast tumor cells.

#### 2.2.2. Non-Covalent Binding

The non-covalent functionalization of the NPs surfaces is based on the physisorption of biomolecules through low energy interactions of attraction and repulsion. These types of interactions can be easily found in many systems such as hydrogen bonding, electrostatic interactions, electron–donor–acceptor ligand systems and weak interactions (van de Waals or dipole–dipole interaction). Compared to covalent bonds, these systems are sensitive to external stimuli such as temperature and pH changes, as well as light irradiation, among others. Many target molecules can adsorb non-covalently to the surface of NPs through electrostatic interactions. In addition, the functionalization with non-covalent interaction precursor groups avoid the use of different and multiple chemical steps to functionalize the surfaces of the NPs, thus simplifying the purification steps and saving a lot of synthesis time [212].

For instance, Conte et al. [213] presented a nano-platform based on functionalized biodegradable polymeric NPs with an Asn-Gly-Arg (NGR) motifs peptide to detect neuropilin receptor 1 (NRP-1) in breast cancer patients. NRP-1 is a non-tyrosine kinase transmembrane receptor that has been detected in some clinical disorders, overexpressed in endothelial cancer and tumor cells. Biodegradable NPs were based on a diamino terminated PCL in which the NGR peptide was assembled via non-covalent functionalization. In addition, a lipophilic anticancer drug, docetaxel (DTX), was encapsulated into NPs, showing a great potential to be used as an anticancer system.

As we mentioned, external factors such as pH, temperature, and ligands nature can modify non-covalent conjugations. In this sense, Silva and coworkers [214] present a PLGA and PEG-PLGA NPs functionalized with acylated cations by noncovalent interaction. N-terminal acylation promotes peptide interaction with the polymeric surface of the NPs. They studied how the acyl chains, with different lengths, influence the lactoferrin-derived cell-penetrating peptide (CPPS). CPPS take advantage of electrostatic interaction between the positive charges of the cationic amino acid groups with the negative charges of PLGA, thus improving the system. In addition, they demonstrated that the acylated peptide chain is an important step for NPs functionalization without the need to use PEG moiety as a spacer.

## 3. Promising Applications of Nanomembranes and Nanoparticles in the Biomedical Sector

An outline of a few potential uses for NMs and NPs, especially in the biomedical sector, is provided in this section. The review mainly emphasizes the primary areas of application in the field because a comprehensive list would be rather lengthy.

### 3.1. Supported Lipid Bilayers/Proteins

Biological membranes, which are only a few nanometers thick, have a flawless molecular order and are made up of two primary components. Lipids have a structural role by generating a self-assembled continuous bilayer that acts as a diffusion barrier, kept together by hydrophobic interactions. Membrane proteins such as transmembrane proteins and peripheral membrane proteins, which are embedded or transiently connected with the lipid bilayer, are important components of cell metabolism such as exchange and biocatalysis processes. They play a role in cell–cell communication, signal transduction, and ion and nutrient transport. Hence, they are a preferred target for biomedical applications. Because of the intricacy of biological membranes and their interactions with intracellular and extracellular networks, direct research is challenging, and in situ investigations of transmembrane proteins are particularly limited [215]. A variety of biomimetic membranes have been created to solve this problem, with the goal of simulating the basic functionalities of a cell membrane and providing platforms for the systematic investigation of various membrane-related activities. Solid-supported membranes [216], polymer-cushioned membranes [217], hybrid bilayer lipid membranes [218], free-standing lipid layer or suspended-lipid bilayers [219], and tethered bilayer lipid membranes [216,220,221] are all examples of these models.

Lipid surface characteristics such as charge and substrate type (hydrophilic/hydrophobic) influence how they self-assemble on the polymeric substrate. Lipid self-assembly occurs on polymer surfaces primarily as a result of electrostatic attraction and hydrophobic interactions. Charged vesicles can be electrostatically attracted to oppositely charged polymeric NPs by incorporating anionic or cationic lipids into a phospholipid bilayer [222]. In order to lower the system’s free energy, neutral phospholipids such as phosphatidylcholine and dipalmitoyl phosphatidylcholine self-assemble onto hydrophobic polymeric surfaces. In accordance with this concept, the surface functionalization of PLGA NPs using lipids has shown promising results in the creation of PLGA-based clinical nanomedicines. Enhancing the target specificity of the carrier through surface engineering with various lipids also boosts its physicochemical characteristics and NP-cell interactions such as cellular membrane permeability, immunological responses, and long circulation half-life in vivo [223].

**Figure 4:**
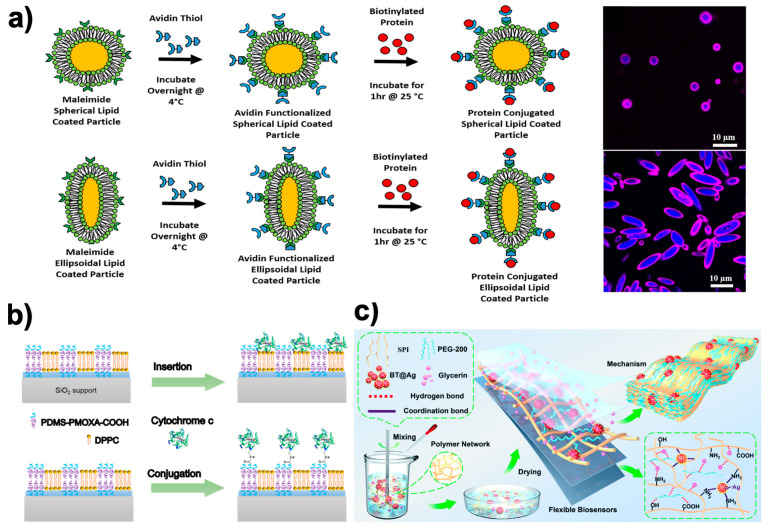
Examples of NMs and NPs as used as lipid-anchored bilayers/proteins and as a biosensor in the biomedical field adapted from various sources. (**a**) Modular protein conjugation to the surface of lipid-coated particles may be made possible by bioorthogonal ligation reactions that are amenable to adaptation. Figure adapted from ref. [224]. Copyright 2018 Elsevier; (**b**) Hybrid-supported bilayer and their combination with model protein cytochrome c through both insertion and covalent conjugation to avoid biosensor protein denaturation after its attachment to solid substrates. Figure adapted from ref. [226]. Copyright 2020 American Chemical Society. (**c**) Schematic fabrication of a highly conductive wearable strain biosensor made of SPI-BT@Ag NM. Figure adapted from ref. [227]. Copyright 2022 Royal Society of Chemistry.

According to Meyer et al. [224], adaptable bioorthogonal ligation reactions may enable modular protein conjugation to the lipid-coated particle surface. This work developed a way to incorporate biologically relevant proteins on a fluidic synthetic lipid membrane with a predetermined anisotropic shape by combining manufacturing techniques for biodegradable particle creation, thin film stretching, lipid coating, and flexible biomolecular conjugation (Figure 4a). This protein presentation can be supported by particles of various shapes and variable radius of curvature, and it replicates the dynamic membrane characteristics of real cells. With the display of laterally movable proteins on the surface of anisotropic biodegradable particles, allowing independent control of the particle’s geometry and permitting the encapsulation of biological cargos, this biotechnology can enable more exact mimicking of natural cells [225].

### 3.2. Biosensors

The development of biosensors for the detection of either biomarkers or states of movement of the human body using nanostructures, either by means of membrane composites and/or NPs with electrochemical properties, has aroused much interest. The high surface-to-mass ratio of these nanostructures provides larger areas of interaction with the biomolecules to be detected, thus improving the sensitivity of the final device. The use of natural polymers with their high biodegradability and biocompatibility, together with their ability to immobilize enzymes and other conductive materials, makes them often used in the integration of biosensors.

Flexible biosensor in point-of-care healthcare applications for the detection of various proteins and biomarkers is a constantly growing field. An important added value is that offered by Xu et al. [228], who reported a flexible, fully organic, biodegradable, biocompatible impedimetric biosensor to monitor vascular endothelial growth factor (VEGF). For this, a conductive ink consisting of a photoreactive silk sericin coupled with a conductive polymer was fixed by photolithography on a flexible free-standing fibroin substrate. Detection was performed through the antibody against VEGF, which was immobilized within the conductive matrix. The biosensor was shown to be highly sensitive and selective for VEGF, even in challenging biofluids such as human serum. However, the biosensoring membranes are likely free-standing but supported on a skeleton device to facilitate their manipulation depending on their final use. For instance, a novel electrochemical biosensor for the detection of human epidermal growth factor receptor 2 (HER2), a breast cancer biomarker was presented [229]. This was shown to be very feasible for use in complex biological samples and human serum with quite acceptable precision. Here, the antifouling detection interface is based on the conductive polymer PEDOT and a biocompatible peptide hydrogel. The peptide hydrogel was prepared from an engineered short peptide of Phe-Glu-Lys-Phe and functionalized with a fluorene methoxycarbonyl group (Fmoc-FEKF) in order to preserve the activity of the immobilized anti-HER2 antibody molecules and their good hydrophilicity. This facilitated effective alleviation of nonspecific adsorption or biofouling, thus extending its functionality.

On the other hand, the stability and activity of complex proteins used as a biosensor molecule can be greatly compromised by the way it is immobilized within a foreign environment. Thus, new methodologies that try to mimic the natural environment of these proteins are emerging. It has recently been shown that lipid, polymeric, and hybrid planar membranes based on mixtures of lipids and copolymers on a solid support provide a more favorable environment for driving the selective and functional binding of a model redox protein such as cytochrome c (cyt c) (Figure 4b) [226]. In this case, the authors showed how polymer membranes provide sufficient chemical versatility to support covalent binding with protein (cyt c), while lipid membranes provide the flexibility and biocompatibility that facilitate the insertion of the protein (cyt c) through its hydrophobic part. This example showed how hybrid membranes combine the most promising features of lipids and polymers and enable the binding of cyt c with covalent binding as well as its insertion driven by hydrophobic interactions. Indeed, these results support the development of complex and versatile hybrid bio-interfaces containing both lipid and polymer domains.

Nanoparticle size allows a better diffusion inside biological systems. Customized biofunctionalization methods can transform inert NPs into hybrid nanosystems capable of targeting specific receptors that trigger specific cellular responses dictated by proteins located on their coating layer. Navarro–Palomares et al. [230] were able to mimic the specific cellular entry mechanisms of certain natural ligands. In fact, they were able to reproduce the molecular cues found in Shiga toxin to target the biosensing NPs into head and neck cancer (HNC) cells bearing the globotriaosylceramide receptor (GB3). To achieve functional biomimetics, they coated SiO_2_ and Fe_3_O_4_@SiO_2_ NPs with a recombinant chimeric protein containing the harmless B-domain of Shiga toxin genetically fused to a nanomaterial-binding sequence. The entry of biosensor NPs into tumor cells allows for better identification and/or subsequent treatment.

Nevertheless, sensor NPs are usually found immobilized within a polymeric matrix to give them mechanical support or to reinforce their biosensor character. Recently, a new biosensor that can potentially be used for the determination of L-asparagine in the diagnosis and treatment of leukemia has been proposed [231]. It is an electrochemical biosensor based on chitosan alginate (CA) NPs that contain L-asparaginase trapped inside. The NPs were deposited on a nylon membrane that covered an ion sensitive electrode (ISE) to detect the ammonium ions (NH_4_^+^) emitted in the reaction of L-asparagine with L-asparaginase. Furthermore, a promising strategy in the development of a new generation of multifunctional flexible wearable biosensors can be seen in the work proposed by Wei et al. [227]. Specifically, a combination of strawberry-type BaTiO_3_ (BT) inorganic particles, soy protein isolate (SPI) chains, polyethylene glycol-200 (PEG-200), and glycerin (GL) to fabricate an SPI-based membrane material (SPI-BT@Ag0.5) was proposed (Figure 4c). This material simultaneously exhibits outstanding yield strength (37.6 MPa), toughness (19.0 MJ m^−3^), and fatigue strength, as well as being low-cost, easy-to-process, and highly conductive. Successful monitoring of human physiological signals and motion states in combination with excellent biocompatibility and biodegradability were reported.

### 3.3. Selective Drug Delivery

Promising biodegradable and biocompatible delivery systems for medications and other therapeutic agents are being tested in many research projects using biopolymeric nanomaterials of various origins. The experiments sought to increase the loaded cargo’s bioavailability and therapeutic impact while also guarding against any potential negative side effects. Bioactive substances such as polyphenols, conventional medications such as those used to treat cancer, antibiotics, natural extracts, and essential oils are just a few examples of the various sorts of cargo being loaded [232]. They are chemically unstable as a result of their susceptibility to external factors such as light, heat, and oxygen. Moreover, they are rapidly metabolized and have limited water solubility. By integrating bioactive substances into nanocarriers such as biopolymeric nanomaterials, these restrictions can be effectively addressed. They can also lower the dosage of bioactive substances needed, lowering the risk of additional negative effects [233]. Here, the emphasis is on the most up-to-date toolkit of literature.

Combinational therapeutic strategies using chitosan (CS), ampicillin (AMP), and pegylated-ZnO (P-ZnO) NPs were developed to increase antibacterial efficacy against infections that produce extended-spectrum beta-lactamases (ESBLs) and are antibiotic-resistant [234]. To ensure the efficacy of the biological qualities, AMP was first loaded onto the fully manufactured CS NPs using adsorption methods in this study. This was followed by a coating with P-ZnO NPs. It was discovered that CS-AMP-P-ZnO NPs had greater antibacterial activity than CS-AMP and AMP alone. Additionally, it was shown using cell membrane potential, protein leakage, and biofilm inhibition assays that the combinational treatment strategies greatly increased the antibacterial and biofilm inhibition efficacy by changing the permeability of the cell membrane. This study implies that the developed combinational antibacterial medication may be a possible treatment for several bacterial illnesses.

**Figure 5:**
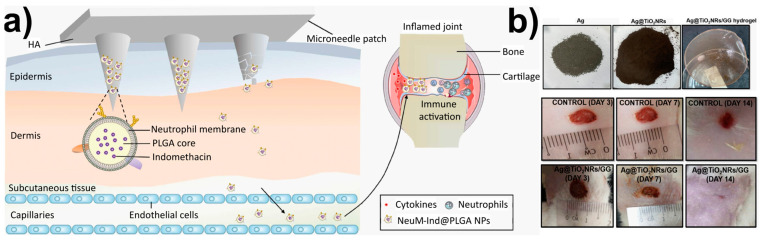
(**a**) Delivery of neutrophil membrane encapsulated NPs by degradable polymer microneedle patch for rheumatoid arthritis therapy. Figure adapted from ref. [235]. Copyright 2023 Elsevier; (**b**) Wound healing application. Macroscopic examination of untreated and treated wounds with Ag@TiO_2_NRs/GG hydrogel film on day 3, 7, and 14. Figure adapted from ref. [237]. Copyright 2023 IOP Publishing.

Nanoparticles have long been the focus of attention in the biomaterial world as a medicine delivery mechanism. A new paradigm of biomaterials with a wide range of applications based on prolonged drug release is made possible by the incorporation of NPs into other biodegradable platforms such as patches, implants, and films. Lin et al. [235] described the delivery of non-steroidal anti-inflammatory drugs (NSAIDs) by degradable biopolymer microneedle patch for the treatment of rheumatoid arthritis (Figure 5a). The neutrophil membrane (NeuM) was used to coat the polymeric NPs that contained the NSAIDs in this dual drug delivery patch before they were inserted into the microneedle patch for local transdermal delivery. The microneedle patch progressively dissolves and releases the encapsulated NPs when used on murine models. The surface modification of PLGA NPs by NeuM is anticipated to mimic the source cell neutrophil, boosting the inflammatory joint targeting and cytokine adsorption of the NPs. The hyaluronic acid microneedle is intended to improve the transdermal absorption of indomethacin. This work offers a potential combination approach to treating rheumatoid arthritis clinically, which may potentially widen the approach to treating autoimmune disorders with anti-inflammatory drugs.

Additionally gaining popularity as a potential drug release platform are polymeric thin films. Prodigiosin (PG), a bacterial pigment with potent anticancer properties, was coupled with poly(3-hydroxybutyrate-co-3-hydroxyvalerate) (PHBV) to create functionally improved PHBV-based biomaterials [236]. The acquired samples were made using a straightforward solvent casting technique, and had a film thickness ranging from 115.6 to 118.8 µm. Moreover, the films were cytotoxic to colon cancer cells, demonstrating the potential for the PHBV/PG biomaterials to be employed in anticancer therapy.

### 3.4. Wound Dressing and Skin Regeneration

To promote wound healing, the primary purpose of wound dressings is to provide a moist, hospitable environment. The following characteristics are necessary for a dressing to be effective: Wound dressings should (i) be compatible with living tissue, (ii) non-toxic, (iii) compliant with tissue, (iv) permeable to water vapor, (v) able to absorb water, (vi) have sufficient mechanical characteristics, and (vii) shield the wound from contamination [238,239]. Researchers in biomedical engineering have recently focused heavily on biodegradable-based dressings made up of natural and synthetic materials for effective and speedy management of wound damage along with lowering the bacterial infection rate. This biopolymeric-based technology has the ability to overcome all the drawbacks of auto- and allografting, and is thought to have a fantastic approach to the process of skin repair and regeneration [240].

Film, foam, gel, and hydrocolloid are just a few of the modern forms that wound dressings can take. The films’ flexibility allows them to conform to the shape of the patient’s body, even in tight spaces such as around the joints, allowing the medication to be applied directly to the wound. The films are gas permeable and prevent bacteria from entering the wound site [241,242]. In light of this, a gellan gum (GG)-based hydrogel film (Ag@TiO_2_NRs/GG) containing Ag-loaded TiO_2_ nanorods (TiO_2_ NRs) was recently published [237] (Figure 5b). TiO_2_NRs, which have an elongated structure and are considered cutting-edge one-dimensional fillers, were utilized in the manufacturing process of hydrogel film. As a secondary form of reinforcement, the Ag particles are incorporated into the hydrogel film in an effort to improve its properties. The presence of Ag in conjunction with TiO_2_NRs fosters both the viability and proliferation of cells. By the fourteenth day after receiving treatment with Ag@TiO_2_NRs/GG hydrogel film, wounds on rats had totally healed. An ultrasound scan of the treated skin reveals an increase in the thickness of all three layers of the epidermis, the dermis, and the subcutis, which is evidence of successful regeneration of the skin’s tissue. Because hydrogels are the most commonly used materials for 4D bioprinting in tissue engineering, the authors decided to employ them for the purpose of achieving on-demand features such as controlled movement, programmable shape alterations, on-demand conveyance, or deterioration.

The utilization of herbal biogenic materials is one inventive method for treating wound infections because of the minimal toxicity and side effects linked with them. Recent years have seen a proliferation of publications in the literature that take this approach. Honey’s naturally occurring antibacterial properties are put to good use in a novel wound dressing material that is composed of gellan gum (GG) and guar gum (GGu) biopolymeric film [243]. The films were made using the solvent casting method, demonstrating the impact of increasing the honey concentration in the system. Enhanced honey concentration in the polymer matrix reduced the swelling capacity of the wound dressing films and enhanced the degradation of the films. Films with a low water vapor transmission rate (WVTR) were appropriate for use as bandages on wounds, while an increase in honey concentration resulted in a higher WVTR. The GG/GGu-H-2.5 film shows promise as a material for efficient wound dressing applications based on evaluations of its mechanical strength, antioxidant characteristics, and wound healing qualities. This novel wound dressing material is an interesting addition to the toolbox. 

Similarly, Sharma et al. [244] fabricated nanoemulsions and film dressings containing thymol in a chitosan-Aloe vera matrix, designed to be readily removed. The film’s ability to absorb water, a vital component in the healing process, was enhanced by the addition of Aloe vera gel. The incorporation of this gel in a wound scaffold, with its anti-inflammatory, anti-tumorigenic, anti-diabetic, and antibacterial capabilities, shall prove to be an integral component in effective healing. The effectiveness and stability of the resultant formulations were verified by their excellent particle size, encapsulation efficiency, antioxidant activity, and in vitro release efficiency. Additionally, several bacterial strains were examined for antibacterial activity. The films were effective in preventing bacterial colonization in a concentration-dependent manner with thymol. Furthermore, Turmeric [245] and bee extracts [246] of varying concentrations were also loaded into dressings made from a combination of non-toxic, biocompatible, biodegradable, renewable, and cost-effective polysaccharide- and protein-based biopolymers. The dressings were designed to prevent bacterial infections without antibiotics, reduce oxidative stress and inflammation, and support wound healing simultaneously.

### 3.5. Actively Targeted Chemotherapeutics

Chemotherapy enhancement has seen widespread use of biodegradable nanoplatforms to avoid the extensive use of chemotherapeutic chemicals that cause damage to healthy tissues. Furthermore, its prolonged use results in systemic toxicity and unfavorable consequences that significantly reduce the maximum tolerable dose of anticancer drugs, and hence reduce their therapeutic efficacy. Most solutions focus on creating highly efficient drug delivery systems that precisely deliver drugs to tumor locations, improving chemotherapy effectiveness while reducing damage to normal cells. Specific drug delivery is based on endogenous or external stimuli such as temperature, light, pH, acidic and reductive conditions, etc. [247,248].

Many nanostructures such as vesicular, dendritic, hydrogel, polymeric, and composite NPs have been developed as drug delivery systems to encapsulated agents (e.g., chemotherapeutic drugs) and their delivery to specific targets. To minimize the risk factors, the research groups are focused on working with resorbable polymers, which can be reabsorbed into the body after the treatment without any harmful products [249]. Polymeric NPs present the optimal properties to be used as a chemotherapeutic platform since they can regulate and transport the drug to cells avoiding the damage of other healthful cells.

**Figure 6:**
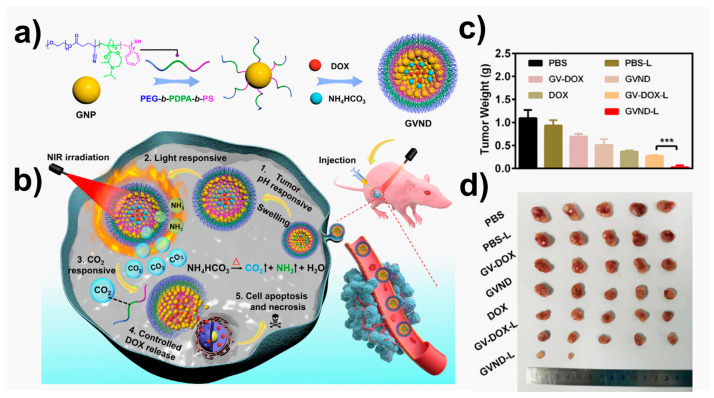
Multi-stimuli responsive gold vesicles (GVND) for programmed release of doxorubicin drug (DOX) against glioblastoma. (**a**) Schematic diagram of GVND preparation and (**b**) their release process; (**c**) changes in the tumor weight of U87MG tumor-bearing mice after different treatments (*** *p* < 0.001); (**d**) pictures of tumor sections collected from different groups after two weeks of treatment. Figure adapted from ref. [251]. Copyright 2023 Elsevier.

For instance, Zhang and coworkers [250] synthesized conjugated polymer-based pH sensitive for synergistic chemo-/photodynamic anticancer therapy. This acid sensitive platform allows the anticancer drug doxorubicin (DOX) to be covalently linked to the side chain of the polymer, improving the stability of the drug delivery system and reducing an early drug leakage. The electronic transfer between DOX and the conjugated polymer backbone effectively were monitored by measuring the fluorescence emission intensity of the poly(fluorene ethynylene) (PFE) backbone. In vitro tests confirmed that aryl-hydrazone linkers of DOX penetrated the cell creating a rupture of the tumor’s intracellular, releasing the drug. Another example of chemotherapy based on external stimuli was recently published by Younis et al. [251] in 2023. In that case, they used near infrared lasers (NIR-lasers) to activate the chain reactions that allow DOX to be released in tumor tissues. Triblock copolymer PEG-*b*-PDPA-*b*-PS, grafted with plasmonic gold vesicles (GVND), were fabricated (Figure 6). This platform offered a precise delivery of chemotherapeutic drugs to tumor tissues under NIR-laser irradiation for mild hyperthermia against glioblastoma. The mild hyperthermia produced by GVND promoted the thermal breakdown of encapsulated NH_4_HCO_3_, leading to the formation of gaseous CO_2_ bubbles in situ. This was made possible by the plasmonic heating of GVND under NIR-laser irradiation. The produced CO_2_ bubbles destabilize the GVND’s vesicular structure, leading to a DOX release in tumor tissues. The system was successfully tested in vitro and in vivo experiments. It is important to highlight that, in such a type of NIR irradiation therapies, the time and power of the source should be well controlled to avoid tissue damages.

### 3.6. Imaging and Diagnostics

Bioimaging is a very powerful and interesting technique that has been developed thanks to its ability to monitor biological processes, which means that diseases can be detected and controlled in vivo. Several methods have been created and developed to obtain accurate images of tumors, cells, bacteria, etc. Among them, the most widely used methods are magnetic resonance imaging (MRI), computed tomography (CT), ultrasound imaging, and positron emission tomography (PET). In the deployment of these methodologies, polymeric platforms have been used because, compared to other materials, they present some important advantages to highlight such as photostability, biocompatibility. and bioresorption.

For instance, Geng et al. [252] synthesized polymer NPs using poly (DL-lactide-co-glycolide) as an encapsulation matrix and PVA as an emulsifier. The fluorogen 2,3-bis(4-(phenyl(4-(1,2,2-triphenylvinyl)-phenyl)amino)-phenyl)-fumaronitrile(TPETPAFN) was encapsulated on that matrix. A co-encapsulation matrix based on poly([lactide-co-glycolide]-b-folate [ethylene glycol]) (PLGA-PEG-folate). NPs surfaces were functionalized with folic groups, which were applied for targeted cellular imaging.

Another well-known technique used in bioimaging is fluorescence microscopy, featuring highly efficient light collection, multiple emissive wavelengths, ease of modification, photostability, and low cytotoxicity. In 2021, Ueya and coworkers [253] encapsulated a red dye (IR-1061) in polymeric micellar NPs formed from poly(ethylene glycol)-*b*-poly(lactide-co-glycolide). It is interesting to note that PEG-b-PLGA forms a stable polymeric micellar nanoparticle in an aqueous environment (denoted as OTN-PNP). A facile synthesis based on nanoprecipitation was performed and the photostability was enhanced by adding an organic fluorophore, di(thiophene-2-yl)-diketopyrrolopyrrole (DPP) encapsulated within OTN-PNPs. The influence of the molecular weight of the copolymers on the stability of the polymer micellar NPs in aqueous media and the emission intensity of IR-1061 in the polymer NP were studied. In this study, it was concluded that OTN-PNPs with a higher molecular weight of PLGA cores showed higher emission and stability under physiological conditions.

Theranostic nanomedicine is recently emerging as a novel treatment that combines therapeutic and imaging functionalities in a single platform. In recent years, many research efforts have been invested in achieving this objective. The resulting nanosystems, capable of diagnostics, drug delivery, and therapeutic response monitoring, are expected to play an important role in the emerging field of customized medicine [254]. The fact that many nanoplatforms are already imaging agents makes it easy to build these new agents with additional built-in functionality. Its surface chemistry allows it to easily incorporate pharmaceuticals and advertise them as theranostic nanosystems. Nanoparticle-based theranostics, a descendant of the two aforementioned approaches, is still in the early stages of development. Nanoparticle-based imaging and therapy are struggling to make it to clinical trials. However, nanoparticle-based theranostics has already gained interest due to the impetus provided by advances in nanotechnology and the need for more individualized therapy. Some research groups have already been working on different NPs such as silicon NPs, quantum dots (QDs), or gold NPs [255]. In addition, various types of biodegradable polymeric theranostic nanoplatforms are being developed such as fluorescence imaging, polymer-based super-paramagnetic NPs, iron oxide NPs, ultrasound-triggered polymeric NPs, and polymer NPs bearing radionuclides [256]. However, in this review. we will focus on some examples of theranostic platforms based on bioresorbable polymers.

For instance, Saxena et al. [257] presented a new enzymatically biodegradable theranostic fluorescence resonance energy transfer (FRET) probe based on biodegradable L-amino acid polyesters using curcumin (CUR), as a fluorophore donor, and Nile red (NR) dye as acceptor. Functionalized NPs were used as a bioresource platform for bioimaging in breast cancer cell lines. Absorption, fluorescence, and lifetime decays were performed to test the FRET energy transfer between the CUR and NR dye, which present an efficiency of 88% and a Förster distance between donor-acceptor of 27.62 Å and 20.68 Å, respectively (The Förster distance represents the donor-to-acceptor separation at which energy transfer efficiency is 50%). This system is of interest as a theranostic platform since CUR can be used as a killing agent in MCF 7 cells and a bioimaging platform through the FRET probe at the cellular level.

**Figure 7:**
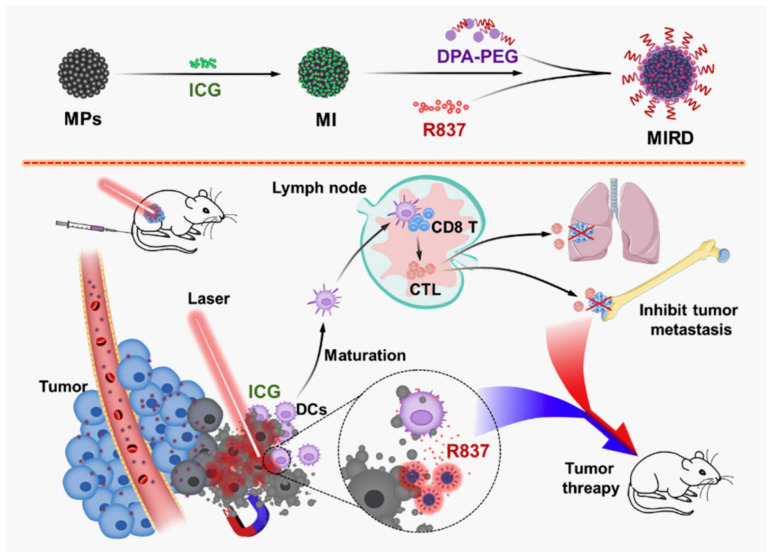
Example of magnetic theranostic techniques for photothermal theraphy/immunotherapy against cancer. Magnetic NPs (MNPs) were used to load indocyanine green (ICG) and immunostimulator R837 hydrochloride (R837) for cancer treatment. Adapted from ref. [258]. Copyright 2020 Elsevier.

Another approach to use polymer NPs for as a theranostic platform is via magnetic nanoparticles (MNPs) composed of iron, cobalt, nickel and their oxides (Figure 7). The most used MNPs are γ-Fe_2_O_3_ (maghemite) or Fe_3_O_4_ (magnetite)] and gadolinium (chelated organic gadolinium complexes). Polymer-based magnetic NP platforms have gained interest for diagnostics and targeted drug delivery because of their sensitivity towards external magnetic fields and their usefulness regarding magnetic resonance imaging [258].

Lazaro–Carillo et al. [259] reported the synthesis of PEG-coated iron oxide NPs (OD15-P5) with a final size of 100 nm to be used as an agent of contrast in magnetic resonance imaging. The nanoplatform was tested both in vivo and in vitro. Throughout the study, the new NPs obtained were compared with Ferumoxytol, a known iron oxide NP approved by the FDA. Both in vivo and in vitro along the magnetic resonance imaging tests, the OD15-P5 NPs obtained showed higher r2 relaxation values than did the ferumoxytol NPs. In addition, a longer residence time of OD15-P5 NPs was observed in the breast cancer tumour cells up to 24 h, whilst ferumoxytol was washed out after 24 h. The results confirmed the great potential of this technology against tumour and cancerous diseases.

## 4. Conclusions and Future Outlook

In the present review, different functionalization mechanisms of NMs and NPs made of natural and synthetic bioresorbable polymers have been discussed. The set of medical applications revisited along this work have provided a glimpse on its great application potential in the near future. Indeed, NMs and NPs have been extensively used in their functionalized form to improve their capabilities and applicability in the fields of supported lipid bilayers/proteins, biosensors, selective drug delivery, targeted chemotherapeutics, and imaging and diagnostics. Recent applications within these specialized fields of biomedicine have been reported, thus showing the impressive adaptability of systems formed by functionalized NMs and NPs.

The exceptional characteristics known of functionalized NMs such as selectivity, high flexibility, stability, robustness, and molecular permeability along with tunable pore size, shape, and densities are giving them a great additional value to be easily adjusted to any specific application. One of the most promising applications in the NMs functionalization field is as a support of lipid bilayers to stabilize large supramolecular structures, simulating the basic functionalities of a cell membrane. This opens a wide variety of bioinspired applications to understand and mimic membrane-related activities [260].

The evolution of the functionalization of NPs and NMs is constantly growing and new ideas are emerging with great potential for use. A few years ago, an interesting proposal for the active functionalization of surfaces emerged from the Zink and Stoddart group [261]. The use of the mesoporous of silica NPs was proposed to store drugs that were retained by means of a chemical gate that closed the pore and that could open depending on certain external stimuli. For example, by controlling the degree of oxidation of the rotaxane that acted as a nanogate and allowing the drug to be released at will. These types of surface nanotools, still under development, may become an interesting drug delivery methodology in the future. Not only responding to redox stimuli but also to pH changes that induce the opening of surface nanogates [262].

On the other hand, NPs systems have been observed to have a large specific surface area, high adsorption capacity, surface reaction ability, and ease of polymerization method application, which lead to a diversity of application in biomedicine. However, the difficulty in isolating the particles and obtaining a homogeneous size distribution is one of the most important drawbacks that can hinder the reproducibility and specificity of those applications where size is an important factor that can affect the biosensing, imaging and diagnostics responses, among other things.

Unfortunately, in the area of nanomaterials, biocompatibility and toxicity remain an open topic that still need to a lot of attention. Physicochemical properties such as size, surface chemical functionalization, shape, protein absorption, and surface morphology play a crucial role in determining the toxicity of nanomaterials such as NMs and NPs. Therefore, considerable additional effort needs to be invested to further study the physiological effects of chronic exposure to nanomaterials in general [263]. NPs are currently used in many biomedical applications. However, cytotoxic effects on cells and organs have to be taken into account as a limiting factor hindering their more extensive use in clinics or hospitals [264].

In addition, NM structures, as with NPs, can be used in various biomedical branches. However, the biocompatibility and toxicity of NMs are not fully understood yet. The use of NM, which is made mostly of natural biomaterials, is helpful to its biomedical applicability due to its low toxicity and low chronic inflammatory responses compared to synthetic ones. Regretfully, owing to the large number of different nanomaterials in use, long-term toxicity studies and tolerability analyzes in animals are still scarce. This is a necessary step before being able to go further and carry out clinical trials in humans [265]. Bioresorbable functionalized NMs and NPs present a very promising future since they combine all the desired properties in implantable or wearable systems that are currently being developed. In fact, their high adaptability will be the key to their extensive use in the field of modern biomedicine.

## Data Availability

Not applicable.
